# Content Analysis of Nicotine Poisoning (Nic Sick) Videos on TikTok: Retrospective Observational Infodemiology Study

**DOI:** 10.2196/34050

**Published:** 2022-03-30

**Authors:** Vidya Purushothaman, Tiana McMann, Matthew Nali, Zhuoran Li, Raphael Cuomo, Tim K Mackey

**Affiliations:** 1 Global Health Policy and Data Institute San Diego, CA United States; 2 Department of Anesthesiology and Infectious Diseases and Global Public Health University of California San Diego La Jolla, CA United States; 3 Department of Anthropology University of California San Diego La Jolla, CA United States; 4 S-3 Research San Diego, CA United States

**Keywords:** nic sick, vaping, tobacco, social media, TikTok, content analysis, smoking, nicotine, e-cigarette, adverse effects, public health, infodemiology

## Abstract

**Background:**

TikTok is a microvideo social media platform currently experiencing rapid growth and with 60% of its monthly users between the ages of 16 and 24 years. Increased exposure to e-cigarette content on social media may influence patterns of use, including the risk of overconsumption and possible nicotine poisoning, when users engage in trending challenges online. However, there is limited research assessing the characteristics of nicotine poisoning–related content posted on social media.

**Objective:**

We aimed to assess the characteristics of content on TikTok that is associated with a popular nicotine poisoning–related hashtag.

**Methods:**

We collected TikTok posts associated with the hashtag #nicsick, using a Python programming package (Selenium) and used an inductive coding approach to analyze video content and characteristics of interest. Videos were manually annotated to generate a codebook of the nicotine sickness–related themes. Statistical analysis was used to compare user engagement characteristics and video length in content with and without active nicotine sickness TikTok topics.

**Results:**

A total of 132 TikTok videos associated with the hashtag #nicsick were manually coded, with 52.3% (69/132) identified as discussing firsthand and secondhand reports of suspected nicotine poisoning symptoms and experiences. More than one-third of nicotine poisoning–related content (26/69, 37.68%) portrayed active vaping by users, which included content with vaping behavior such as vaping tricks and overconsumption, and 43% (30/69) of recorded users self-reported experiencing nicotine sickness, poisoning, or adverse events such as vomiting following nicotine consumption. The average follower count of users posting content related to nicotine sickness was significantly higher than that for users posting content unrelated to nicotine sickness (*W*=2350.5, *P*=.03).

**Conclusions:**

TikTok users openly discuss experiences, both firsthand and secondhand, with nicotine adverse events via the #nicsick hashtag including reports of overconsumption resulting in sickness. These study results suggest that there is a need to assess the utility of digital surveillance on emerging social media platforms for vaping adverse events, particularly on sites popular among youth and young adults. As vaping product use-patterns continue to evolve, digital adverse event detection likely represents an important tool to supplement traditional methods of public health surveillance (such as poison control center prevalence numbers).

## Introduction

TikTok is a social media platform that has gained millions of followers and now has brand recognition comparable to that of other well-known social media platforms, such as Facebook, YouTube, and Twitter [1]. TikTok users post, share, and rank microvideos or short clips (between 15 seconds to 3 minutes in duration), with the potential for their content to go viral depending on the level of user interactions, shares, hashtags, and subject matter [2]. In 2021, TikTok reported 1.2 billion monthly active users, and 1.5 billion monthly active users are projected for the end of 2022 [1,3]. It was one of the fastest growing social media platforms, with over 2 billion total downloads since its launch [3]. Approximately 60% of monthly TikTok users in the United States are between the ages of 16 and 24 years [4]—a demographic that is at risk for tobacco initiation and uptake [5]. Additionally, the platform has also been identified as a source of pro–tobacco and tobacco product sentiment [6,7].

Uptake of electronic nicotine delivery systems, as well as patterns of use, can be impacted by social interactions that occur in digital environments as a result of exposure to marketing content, influencers, and other pro–nicotine product sentiment shared by users [8,9]. Increased social media use among adolescents can lead to increased willingness and intention to use e-cigarettes, with reported positive and normative perceptions [10]. Concurrent with the rise in popularity and high rate of adoption among youth and young adults on emerging social media platforms such as TikTok, the increased popularity and use of electronic nicotine delivery systems has given rise to a vaping epidemic [11,12]. For example, from 2016 to 2018, there was a 2.9% increase in smoking prevalence among young adults aged 18 to 24 years in the United States, and more than one-quarter of 10th graders reported having used an electronic nicotine delivery system product [13]. Due to this appeal, vape manufacturers and web-based sellers reportedly use TikTok influencers and direct marketing and selling of electronic nicotine delivery system products on the platform, although public health and tobacco control advocates have also attempted to use TikTok for health promotions warning about the risks of vaping [14].

One of the consequences of increased use of electronic nicotine delivery systems has been the rise in nicotine-related adverse events and poisoning, which was highlighted by the 2019 outbreak of lung injury associated with use of e-cigarette or vaping products that led to 2807 hospitalizations or deaths in the United States [15]. Nicotine poisoning refers to the toxic effects of nicotine consumption that are beyond an individuals’ tolerance. Poisoning causes nonspecific symptoms such as nausea, vomiting, tremors, and increased heart rate in early phases and severe symptoms such as shock, low blood pressure, and even paralysis in later phases [16]. Complicating this public safety risk, nicotine poisoning and other electronic nicotine delivery systems adverse events have increased [17,18], partially due to the introduction of new nicotine products, some of which still require market authorization by regulatory agencies [17]. Most nicotine poisoning research and product surveillance is based on data from government consumer safety organizations (eg, US National Electronic Injury Surveillance System) or poison control centers [19], with only a few studies [20] using social media surveillance to detect adverse events. *Nic sick* is a term that has been used to refer to these adverse effects by users on various social media platforms who post content related to nicotine sickness [21] and can be used as a keyword to curate user-generated self-reporting about nicotine sickness topics and lived experiences.

While TikTok is a platform that can promote and share beneficial health-related content and possibly mobilize public health campaigns [22-24], it can also spread harmful information [25], including content that promotes the use and overuse of electronic nicotine delivery systems to its large audience of youth and young adults [18,26,27]. For example, recent published studies [28,29] that examined health information videos on TikTok observed that the overall quality of health information can vary based on the source of content and that users should be selective and cautious when viewing such content. Additionally, the findings of systematic thematic analysis in 2021 [7] showed that a majority of electronic nicotine delivery system–related TikTok videos portrayed electronic nicotine delivery system–use positively. Another study [30] found that disposable electronic nicotine delivery system product content was popular among TikTok users. Therefore, due to the growing popularity of electronic nicotine delivery system content on the platform, in addition to the high burden of nicotine consumption and risk of nicotine adverse events among TikTok youth and young adults [31], we aimed to expand on prior research by examining the specific characteristics of nicotine poisoning–related TikTok content.

## Methods

### Ethics

All information used in this study was posted publicly, and the study did not involve any interaction with users. User identifiable information was removed.

### Data Collection

The hashtag #nicsick was originally selected for review based on news and media reports on the popularity of the term for users’ self-reporting experiences and topics associated with nicotine sickness [21]. The selection of #nicsick as the hashtag for data collection was confirmed by conducting preliminary manual searches with the TikTok in-platform search function for keywords, terms, and hashtags that were used in videos discussing nicotine sickness content. In addition, we conducted preliminary Google Trends search terms analysis to assess if there were any queries or topics related to the *nic sick* terminology set for the study period, which did not reveal any specific terms for further keyword or hashtag data filtering. TikTok videos with the hashtag #nicsick were collected retrospectively in May 2021 by first using structured searches on the platform without any user log-in credentials, with no personal search history and with cookies disabled. The URLs and metadata (eg, date, time, username, favorites) associated with retrieved TikTok videos using the hashtag #nicsick were collected and saved using an algorithm (Python, version 3.7.0) and an automated web browser and automation tool (Selenium, version 3.141.0; SeleniumHQ). In addition, the videos were also downloaded and saved for content analysis.

### Data Analysis

Content analysis of TikTok videos collected was conducted by VP, TM, and MN. The videos were manually annotated (using binary classification—whether the video depicted nicotine poisoning or nicotine sickness or not) and then inductively coded for specific themes that emerged, such as showing active vaping, specific adverse events, or nicotine sickness experiences (Table 1). Furthermore, based on prior research studies [7,32,33] that have conducted content analysis on TikTok videos, we also coded the following metrics and associated metadata: (1) user engagement (views, likes, comments, shares, followers, verification); (2) video characteristics (duration, caption, text on screen, subtitles, music); and (3) video type (original, duet, stitch). An *original* video is a microvideo uploaded by a TikTok user and is the primary source of user-generated posts on the platform. A *stitch* integrates the user’s own video into another user's original video by clipping and integrating scenes from the original video which allows the sharing of existing videos in combination with new user-generated content. A *duet* video builds on another user’s original video, with a new video recorded alongside the original as it plays and is mainly used for user reaction videos. VP, TM, and MN coded posts independently and achieved high intercoder reliability (κ=0.96). For inconsistent results, senior author TKM was consulted.

After open inductive coding based on our coding schema, videos with nicotine poisoning content were grouped into main thematic categories: (1) firsthand and secondhand reporting of suspected nicotine sickness symptoms (eg, nausea, headache, cough, stomachache, and regurgitation); (2) videos displaying actual poisoning-related adverse events; and (3) videos displaying active vaping while discussing nicotine sickness–related content. The 2-tailed independent sample *t* test and Wilcoxon rank-sum test were used to compare the engagement metrics of videos with and without active nicotine sickness–related content. All statistical analyses were conducted using RStudio (version 3.6.1). A *P* value<.05 was considered statistically significant.

**Table 1:** Coding scheme for videos.

Theme and subtheme				Coding scheme
**Nicotine sickness symptom reporting (firsthand or secondhand)**				
	Yes			Videos of users discussing specific symptoms related to nicotine sickness, such as vomiting, nausea, burning sensation in throat, headache, and fatigue, during or after nicotine use Users discussing nicotine sickness symptoms of self or friends, family, and neighbors Users discussing clinic/hospital/emergency room visits for alleged nicotine sickness symptoms Videos of users seeking suggestions to overcome nicotine sickness symptoms
	**No**			Posting videos on news posts related to nicotine sickness Videos on nicotine sickness symptoms by users or public health organizations to create awareness about nicotine sickness Videos unrelated to nicotine sickness using the hashtag #nicsick Videos with jokes or sarcasm about nicotine sickness
		Displaying poisoning-related adverse events	Videos with users displaying symptoms related to nicotine sickness or poisoning	
		Actively vaping	Users actively vaping while discussing nicotine sickness–related content in the video	

## Results

A total of 134 TikTok videos with #nicsick were collected, but 2 videos were inaccessible due to user privacy settings and were excluded from analysis. After manual annotation, we confirmed that 52.3% (69/132) of videos included content discussing nicotine sickness, poisoning, or adverse event symptoms, of which 52 were original videos, 16 were stitches, and 1 was a duet. Of these 69 videos, 36.23% (n=25) were posted in the year 2020 and 63.77% (n=44) in 2021; and the earliest was posted on January 12, 2020, and the latest was posted on May 24, 2021. The videos that were unrelated to nicotine sickness–reporting primarily depicted content related to vaping behavior and other topics, but nevertheless, included the hashtag #nicsick.

**Figure 1:**
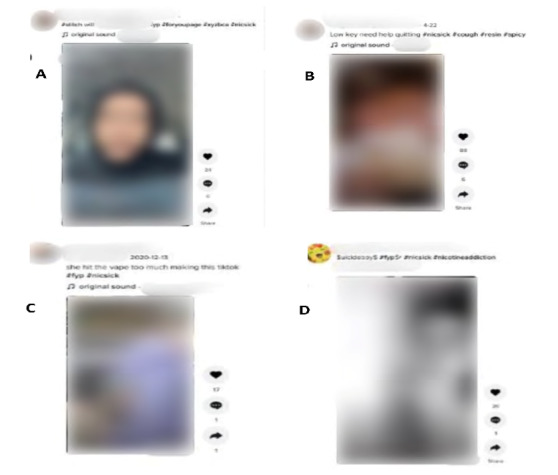
TikTok video screenshots with signal for nicotine sickness (“nicsick”; user information concealed and images blurred).

Videos with content related to nicotine sickness discussed specific symptoms, such as vomiting, nausea, burning sensation in throat, headache, and fatigue, during or after nicotine use. Apart from posting videos with content on nicotine sickness experiences, users also asked for suggestions on how to overcome nicotine sickness symptoms from other TikTok users who may have previously experienced similar adverse effects following nicotine consumption. More than one-third of the videos with nicotine poisoning content (26/69, 37.68%) portrayed active vaping (including extreme vaping behavior such as vaping tricks, nicotine overconsumption, attempts at performing vaping challenges) and 43.5% (30/69) recorded users experiencing nicotine sickness, poisoning, or adverse events, such as vomiting after nicotine consumption. While the first category of videos with only mentioned users experiencing various adverse effects during or after nicotine consumption, the second category of videos recorded the adverse effects, such as vomiting, nasal discharge, vigorous coughing, directly in the video (Table 2; Figure 1).

**Table 2:** Descriptions of #nicsick examples (deidentified).

Theme	Definition	Example
Firsthand (self) and secondhand reporting of nicotine sickness symptoms.	A user reporting one or more suspected nicotine sickness symptoms (nausea, cough, regurgitation, etc) currently or in the past.	Figure 1A is a screenshot of a TikTok video in which the user describes their own experience with nicotine sickness
Users displaying adverse events related to nicotine poisoning.	A user exhibiting one or more adverse events (regurgitation, cough, etc) related to nicotine poisoning.	Figure 1B and Figure 1C is a screenshot of a TikTok video in which the user describes their own experience with nicotine sickness Figure 1B shows a user experiencing nasal discharge. Figure 1C shows user vomiting on the side of the road after making a TikTok.
Users actively vaping	A user actively vaping on video.	Figure 1D is a screenshot of a TikTok video in which the user is seen actively vaping using an electronic nicotine delivery systems device.

A total of 67 TikTok users posted the 69 videos with nicotine sickness content, of which 66 users were unique, while only 1 user (with a follower count of 2487) posted 3 videos, with 2 of them discussing adverse events after nicotine consumption. The 63 videos with content unrelated to nicotine sickness were posted by 60 users, of which 57 user IDs were unique, while 3 users posted 2 videos each. Upon comparing the user IDs, we found that 3 users had posted signal and nonsignal videos using the hashtag #nicsick.

Videos with nicotine sickness content had 4415 mean views, 548 mean likes, 4 mean shares, 16 mean comments, and a mean user follower count of 19,485, and videos unrelated to nicotine sickness had 52,117 mean views, 1958 mean likes, 31 mean shares, 30 mean comments, and a mean user follower count of 8669. The follower count of users posting content related to nicotine sickness was significantly higher (*W*=2350.5; *P*=.03) than that of users posting content unrelated to nicotine sickness. The mean differences for views (*W*=1688.5; *P*=.45), likes (*W*=1780; *P*=.56), shares (*W*=1733; *P*=.36), and comments (*W*=1811.5; *P*=.66) between videos with nicotine sickness content and those without were not statistically significant.

The mean duration of videos with nicotine sickness content was 23.68 seconds (range 4-62 seconds) and the mean duration of videos displaying active vaping (12.23 seconds) was significantly shorter (*P*<.001) than that of videos without active vaping (30.60 seconds). However, videos displaying nicotine sickness symptoms were longer in duration (28.57 seconds) than those not displaying nicotine sickness symptoms (19.92 seconds), albeit the comparison did not meet the threshold set for statistical significance (*P*=.08).

Users who posted first- or secondhand experiences with nicotine poisoning often did not explicitly or implicitly discourage others from engaging in harmful vaping behaviors such as overconsumption. Instead, users posting with the hashtag #nicsick appeared to actively participate in trending vape tricks or challenges. Some of the phrases heard in TikTok videos included “nic-sick check” and “where’s my JUUL” while users intentionally engaged in vaping behavior to generate TikTok trends (ie, how TikTok uses a hashtag or a song to group together viral trends within the app).

## Discussion

### Principal Findings

We found that TikTok users openly discussed their experiences, both firsthand and secondhand, with nicotine poisoning and related adverse events via the #nicsick hashtag. This includes users promoting extreme vaping behavior, discussion of users’ direct experiences with adverse events, and explicit video documentation of adverse events that appear to elicit user attention and reaction. We also found that users were more likely to create original videos as opposed to stitches and duets, which allow users to imbed and combine existing videos into their own TikTok post, which can often result in higher user engagement [34]. User engagement metrics of the videos in our study indicated moderate and lower compared to general metrics for popular TikTok videos, although some popular creators have had higher levels of engagement and mean follower counts. Additionally, the duration of videos depicting adverse events or nicotine poisoning symptoms was marginally longer than those that did not, indicating that videos depicting adverse events may need to be longer in order to provide sufficient details on the reported nicotine sickness experiences versus videos that simply reported or portrayed general vaping behavior.

These emerging forms of microvideo-based social interactions have the potential to change ways in which susceptible youth and young adults receive vaping-related behavior cues. For example, phrases heard in TikTok videos in this study included “nic-sick check” and “where’s my JUUL” and were associated with intentional overconsumption of nicotine products that also appeared to increase user engagement. TikTok’s *For You Page* also personalizes content based on user viewing history and can lead to a continuous cycle of exposure to trending vaping videos, including those in this study that portrayed overconsumption. Characteristics of TikTok videos, including use of hashtags, trending sounds, and video duration, may also represent crucial opportunities for the introduction of targeted health education and promotion to warn young users about the documented harms of vaping, particularly if they are already experiencing and self-reporting adverse events [35]

Yet, it is important to note that the impact of these videos on actual knowledge, attitudes, and behaviors of social media users associated with the appeal, uptake, and use of electronic nicotine delivery systems remains unclear. Vaping themes detected in this study seem to demonstrate that users may be promoting harmful vaping practices, which in turn has the potential to encourage overconsumption or dangerous nicotine use behavior. For example, popular internet challenges, including vape challenges, are rapidly circulated through TikTok—some may greatly harm the health of participants or encourage others to engage in similar behavior [25]. However, the motivations of these users to post #nicsick videos and the reason why they may or may not become viral require further study, particularly in the context of how the risk of electronic nicotine delivery system–related adverse events may be increased. We also found that there was no clear indication that users experiencing nicotine sickness were committed to quitting despite their sickness or poisoning, although a few user videos included individuals expressing a desire to quit. Instead, users using #nicsick appeared to be actively participating in trending vape tricks or challenges, which then led to overconsumption followed by one or more adverse events, which they then posted.

Combating the youth vaping epidemic requires scrutiny of how new interactive media, such as TikTok, serves as a source of vaping-related information and a source of peer influence among young user communities. Traditional poisoning and adverse event surveillance systems, such as the National Poison Data System and the US National Electronic Injury Surveillance System, may fail to capture these less severe cases captured on social media platforms, thereby underrepresenting the morbidity burden of nicotine poisoning [19]. Hence, social media platforms such as TikTok, may represent an important data source that can be used to help identify the burden of user-reported nicotine exposure and poisoning.

Our study narrowly focused on identifying and characterizing how nicotine poisoning–related content is created and shared through TikTok’s microvideo posts. Future studies should focus on examining the complex interplay between user exposure and the appeal of TikTok pro–tobacco and vaping content, the dynamics of how this content is shared and propagated on these networks, how it impacts real-world use patterns and vaping behavior, and finally, how these factors may impact safety and health issues, such as adverse events. Though TikTok’s policies state that content which offers the purchase, sale, trade, or solicitation of tobacco products (including vaping products) is prohibited [36]. Our study results indicate that content related to excessive or potentially dangerous smoking or vaping behavior does not fit this criterion, although the utility of removing such content is also unclear.

### Limitations

We used a single platform and a fixed data collection period. Hence, the results may not be representative of all nicotine sickness content on social media. While TikTok is a popular social media platform, the demographics of TikTok users may not reflect those of the general population of electronic nicotine delivery system users. Furthermore, study data were limited to results for videos queried and returned by TikTok’s internal search function, which may further limit generalizability. We used a single hashtag, #nicsick, based on a combination of open-source news reports, manual searches, and a preliminary Google Trends analysis to justify inclusion and due to the narrow focus of the study on nicotine sickness–related content. Hence, this study might have missed capturing additional nicotine sickness content with other hashtags that may have focused on specific electronic nicotine delivery system products, vaping behavior and challenges, and other nicotine and vaping–related topics with content related to nicotine sickness or adverse events. Furthermore, we did not cross-validate user-generated content with other data sources of nicotine sickness reports or cases, such as data from poison control centers, and we did not explore user interaction or social network characteristics of web-based user networks where videos with nicotine poisoning content was shared, viewed, or interacted with, via favorites or comments, in-depth. Future studies should conduct more comprehensive and large-scale data analysis of nicotine-related adverse events on TikTok compared with those on other social media platforms. Future research can explore further analysis of characteristics of videos including hashtags, trending sounds, and video duration, which may help curate targeted health education and promotion against vaping use.

### Conclusion

While we observed discussions and experiences with nicotine poisoning and adverse events on TikTok, further research is needed to assess how this unique social media risk environment impacts user perceptions about the known harms of vaping and which health promotion strategies can be tailored for youth experiencing vaping-related adverse events. Although other electronic nicotine delivery system–promoting content on TikTok may influence tobacco use behavior, #nicsick-specific content represents user-generated experiences directly associated with adverse events or poisoning due to nicotine overconsumption, which could be an alternative form of surveillance to better characterize this likely underreported public health issue. Supplementing traditional surveillance methods with infodemiology and infoveillance approaches can help in capturing the nicotine sickness cases that are not reported to poison control centers and assess the growing health burden due to nicotine use more appropriately.

## Data Availability

Raw data supporting the conclusions of this paper will be made available by the authors, without undue reservation.
